# Allopathic and traditional health practitioners: A reply to Nemutandani, Hendricks and Mulaudzi

**DOI:** 10.4102/phcfm.v9i1.1284

**Published:** 2017-04-26

**Authors:** Rudi W. de Lange

**Affiliations:** 1 Department of Visual Communication, Tshwane University of Technology, South Africa

## Abstract

An earlier paper in this journal reported on the perception and experience of 77 allopathic health practitioners (AHPs) and health managers about working together with South African traditional health practitioners (THPs). The paper stated that the abolishment of the *Witchcraft Suppression Act* of 1957 and the introduction of the *Traditional Health Practitioners Act* No. 22 of 2007 is a milestone in the development of traditional health knowledge, and for the eventual incorporation thereof into modern health care practices. The authors also comment that a decolonisation of mindset and a change of attitude is required to change one’s perception of traditional healer practices and to develop them parallel to allopathic health practice. This opinion paper is a response to the paper, to negate its claims about the *Witchcraft Suppression Act* of 1957 and to provide clarity on the *Traditional Health Practitioners Act* No. 22 of 2007 and related policies and regulations. Although this Act recognises THP, the Act and other regulations actually require THP to conform to practices analogous to those of AHP. It is rather a systematic and scientific ‘mindset’ that is required to develop THP parallel to AHP. The *Traditional Health Practitioners Act* of 2007 and the Draft Policy on African Traditional Medicine (TM) for South Africa dictate that a substantial THP sectoral transformation is required before there can be a parallel system. Legislation and regulations have excluded THP and African TM from operating (present and future) in the same space as AHP.

## Introduction

In a paper that appeared in an earlier edition of this journal, Nemutandani et al.^1^ report on the perception and experience of 77 allopathic health practitioners (AHPs) and health managers about working together with South African traditional health practitioners (THPs). Their respondents’ views are that the health systems are incompatible with regard to the source of knowledge and the basis of their science. AHP and health managers replied that working with THP will inter alia compromise the quality of their health care. The survey further highlights that there is a general lack of knowledge of the Act that regulates THP.

The introduction states that the abolishment of the *Witchcraft Suppression Act* of 1957^2^ and the introduction of the *Traditional Health Practitioners Act* No. 22 of 2007^3^ is a milestone in the development of traditional health knowledge and for the eventual incorporation thereof into modern health care practices. They further state that one needs to ‘embrace the existing cultural diversities, community health practices and belief systems in the South African health-care system’. In addition, they mention that traditional medicine (TM) and its beliefs were outlawed before 1994. They also suggest that a decolonisation of mindset and a change of attitude is required to change one’s perception of traditional healer practices and to develop them parallel to allopathic health practice.

## The aim of this opinion paper

This opinion paper is a response to Nemutandani et al.’s paper, in particular to negate its claims about the *Witchcraft Suppression Act* of 1957, to provide some clarity on the *Traditional Health Practitioners Act* No. 22 of 2007 and related policies and regulations. I argue that although this Act recognises THP, the Act and other regulations actually require THP to conform to practices analogous to those of ‘colonial’ AHP. I further argue that it is rather a systematic and scientific ‘mindset’ that is required to develop THP parallel to AHP, and that proposed regulation will essentially create a more restrictive environment for THP and traditional African medicine than the pre-1994 conditions.

## The *Witchcraft Suppression Act*, No. 3 of 1970

Nemutandani et al. mention that the *Witchcraft Suppression Act* of 1957 has been abolished and that traditional healing practices were repressed through prohibition laws. This Act has not been repealed and is still in force. The Act lists five items as offences. A person is guilty of an offence if such a person (1) credits another person as having supernatural powers to cause ‘any disease in or injury or damage to any person or thing, or who names or indicates any other person as a wizard’; (2) uses a ‘witch-doctor, witch-finder or any other person to name or indicate any person as a wizard’; (3) professes a ‘knowledge of witchcraft, or the use of charms, and who advises any person how to bewitch, injure or damage any person or thing, or who supplies any person with any pretended means of witchcraft’; (4) on the advice of a person’s beliefs takes steps to ‘injure or damage any person or thing’; (5) for gain pretends to have the ability to tell fortunes or to ‘discover where and in what manner anything supposed to have been stolen or lost may be found’. The updated *Witchcraft Suppression Act*, which was amended in 1970 and 1997 (Act 50 of 1970 and Act 33 of 1997), lists six actions that are deemed to be offences. These are the same as the five in the original Act, with the addition of one item, namely that it is an offence if a person claims to have the ability to cause ‘the death of, injury or grief to, disease in, damage to or disappearance of any person or thing to any other person’. The essence of the original and the updated Act is to protect people who are accused of having esoteric abilities and that they are the cause of misfortune that befalls others. The Act is currently under review. The South African Law Reform Commission has produced a draft bill,^4^ the Prohibition of Harmful Practices Associated with Witchcraft Beliefs. This bill provides:

for the prohibition of harmful practices associated with the accusations that a person is a witch; to protect communities from violence often associated with claims about harmful witchcraft practices; to criminalise harmful practices associated with witchcraft beliefs; to provide for penalties for harmful practices identified; and to provide for matters connected therewith (p.92).

I have found sufficient evidence for people being prosecuted for causing harm to others^5^ (in terms of witchcraft) but was not able to trace a case where a person was prosecuted for practising as a traditional healer. I am also not aware of a pre-1994 Act that explicitly outlawed traditional healing practices. However, the national coordinator for the Traditional Healers Organisation^6^ confirmed (Maseko P 2016, personal communication, July 28) during a meeting that this Act is seen and perceived by traditional healers as a repressive instrument.

## Policy documents, legislation and traditional health practitioners

The Department of Health’s Strategic Plan for the Prevention and Control of Non-Communicable Diseases 2013–2017^7^ provides a five-year government strategy to reduce illness and mortality from communicable diseases. The only reference to traditional healers in the strategic plan, is in Appendix 1, where the government states its intention to ‘build links with traditional and complementary healers’. Appendix 2, a political declaration from heads of state, makes only two minor references to traditional and alternative healing practices. Governments are to recognise, preserve and promote TM in accordance with a country’s capacity, legislation and a country’s circumstances.^7^ The South African National Development Plan Vision for 2030^8^ provides inter alia plans for health care and steps that would improve the lives of South Africans. Chapter 10 devotes only one paragraph to traditional healers. The plan acknowledges that there is limited integration with the national health care system and mentions that the Department of Health needs to develop a policy on how to accommodate TM into the health system. The Department of Arts and Culture’s National Policy on South African Living Heritage,^9^ in a similar vein, devotes only one paragraph when discussing the position of traditional healers. They emphasise that practices must be aligned with the Bill of Rights, relevant legislation, and that traditional healers must ensure that that their activities are aligned with ubuntu and the Constitution.

The aim of the *Traditional Health Practitioners Act* No. 22 of 2007 is to establish a council, to provide for the registration and training of THPs and to protect the clients of THP. Even though the council was established in February 2013, they have difficulty in delivering on their mandate, such as assessing a person wishing to register as a traditional healer.^10^ The Act, however, excludes THP from the activities of professional health practitioners as contemplated in the *Pharmacy Act*, 1974; the *Health Professions Act*, 1974; the *Nursing Act*, 1974; the *Allied Health Professions Act*, 1982; or the *Dental Technicians Act*, 1979. The Act also prohibits THP from any other activity not based on their traditional philosophy.^3^ A person wanting to practise as a THP must furthermore be registered, comply with a code of conduct and will be subject to disciplinary steps in the event of misconduct.

The Department of Health recently released regulations, the Traditional Health Practitioners Regulations 2015.^11^ These regulations concern the training and registration of persons wishing to work as a traditional healer in the categories of divination; herbalism; to practise as a traditional birth attendant; and to practise as a traditional surgeon (circumcision). The regulations list the minimum age, training period, registration process and some administrative procedures. A student practitioner, for example, may only register if he or she has attained Adult Basic Education and Training (ABET) 1 or an equivalent from an accredited institution. Educational prerequisites are mandatory for the training of AHPs but may not be helpful to persons who feel ‘called’ to become a THP and are withdrawn from school.

In 2008, the Department of Health published a Draft Policy on African TM for South Africa.^12^ This draft policy is about the institutionalisation of African TM and is to be implemented through registration and regulation of medicines and medicinal products, and the protection of the knowledge, property rights and persons that are involved in this discipline. The policy is about the development of this practice as a distinct, separate system in the formal health sector, and not one that is to be integrated into the science-based medical practice. The policy further refers to issues such as standards for safety, efficacy and quality; scientific research and clinical studies; formal documentation and systematisation of the products; the development of a pharmacopoeia; an industry for the production and processing; and the licensing of providers. This draft policy is an indication that the government’s intention is for TM to operate as a parallel (or alternative) practice. The National Department of Health has recently advertised^13^ a directorship post for Traditional Medicine in the Directorate: Traditional Medicine. The incumbent’s duties will include the development of the department’s strategy on TM, but ‘in accordance with all of the relevant legislation. Registration and issuing of licences for both Practitioners and Products will be done by the Traditional Health Practitioners Council and the Medicine Control Council, respectively’. Reading the draft policy and the duties of a person heading the Directorate for Traditional Medicine, one is forced to conclude that African TM will have to comply with existing legislation, including the *Medicines and Related Substances Control Act*, No. 101 of 1965.^14^ I discussed the policy on African medicine with the acting director of the Directorate: Traditional Medicine. He confirmed (Mbedzi F 2016, personal communication, August 11) that the regulation of African TM will apply only to those products that will be commercialised (requirements for registration of TMs are yet to be determined by the Regulatory Council). He further mentioned that the parallel development of THP does not mean that these services will be available at public health facilities, but rather that the public will be able to choose between going to a public health facility or to a regulated THP in their area.

## Conclusion

The results of Nemutandani et al. are similar to the opinion^15^ and findings of other^16^ scholars. The source of knowledge and basis of science of the two disciplines THP and AHP are so different that they are incompatible. Reading the Traditional Health Practitioners Act of 2007, the Draft Policy on African Traditional Medicine for South Africa and the Traditional Health Practitioners Regulations 2015 suggests that the future of THP and African TM will be highly regulated and subject to similar scientific processes as AHP. THPs must be registered, must receive formal training and are subject to disciplinary procedures. Their commercial treatments must be registered; they must comply with set standards for safety, efficacy and quality; and they will be subject to research and clinical trials. We could have an African TM pharmacopoeia, commercial production processes and licensed providers. The Act, the Regulations and the Draft Policy, when enacted, will require a rigorous, systematic and scientific approach to develop THP and are not reliant on the decolonisation of one’s mind. Embracing the decolonisation of medicine could, as suggested by De Roubaix,^16^ imply a ‘return to traditional ways of healing’, that a ‘THP should eventually be recognised as a fellow professional’ and that ‘space should be created for THPs within current healthcare structures’. This is despite there being a lack of independent, verifiable evidence for the safety and efficacy of TM.^10^ Such a process of decolonising is clearly not compatible with the scientific and systematic tenets advocated by the Draft Policy on African Traditional Medicine for South Africa.

The *Traditional Health Practitioners Act* of 2007 and the Draft Policy on African Traditional Medicine for South Africa dictate that a substantial THP sectoral transformation is required before there can be a parallel system. Even though the Department of Health’s position^17^ is that they have committed ‘to the involvement of traditional healers in official healthcare services’ and they are to ‘strengthen and promote traditional medicine and practice’, it does not imply a system in the same space as AHP. Legislation and regulations have excluded THP and African TM from operating (present and future) in the same space as AHP. A notion that the *Traditional Health Practitioners Act* of 2007, the Traditional Health Practitioners Regulations 2015 and the Draft Policy on Traditional African Medicine for South Africa pave the way for the incorporation of traditional healing and TM into allopathic health care practices (as opposed to a parallel development) may be premature and possibly misguided.
